# iLIVE volunteer study: Volunteer and healthcare professional perceptions of newly developed hospital end-of-life-care volunteer services, in five countries

**DOI:** 10.1177/02692163251328197

**Published:** 2025-05-29

**Authors:** Tamsin McGlinchey, Stephen Mason, Grethe Skorpen Iversen, Dagny Faksvåg Haugen, Inmaculada Ruiz Torreras, Pilar Barnestein Fonseca, Miša Bakan, Berivan Yildiz, Ruthmarijke Smeding, Anne Goossensen, Agnes van der Heide, John Ellershaw

**Affiliations:** 1 Palliative Care Unit, University of Liverpool, UK; 2 Regional Centre of Excellence for Palliative Care, Haukeland University Hospital, Norway; 3 Department of Clinical Medicine K1, University of Bergen, Norway; 4 Cudeca Hospice Foundation, Spain; 5 University Clinic of Pulmonary and Allergic Diseases Golnik, Slovenia; 6 Erasmus MC, University Medical Center Rotterdam, The Netherlands; 7 University of Humanistic Studies, The Netherlands

**Keywords:** Volunteers, palliative care, hospitals, community support, focus groups

## Abstract

**Background:**

Volunteer services that provide direct support to patients receiving palliative and end-of-life care in hospitals are new and developing, but little is known about the use and experience of such services from key stakeholders.

**Aim:**

Explore the perceptions of volunteers, and healthcare professionals, towards newly established hospital end-of-life-care volunteer services, in five countries.

**Design:**

A phenomenological approach was adopted, using focus group interviews. Data were analysed using an adapted framework analysis.

**Setting/participants:**

Acute hospital in-patient units, in five European countries. Participants were recruited if they were: Volunteers from the end-of-life-care volunteer service, or Healthcare professionals working within the wards that the volunteer service is operational.

**Results:**

20 Volunteers and 20 healthcare professionals were recruited. Most participants were female (70%, *n* = 14/65%, *n* = 13). The healthcare professionals included a majority nurses (60%). Three overall themes were generated: (1) Volunteers provided ‘unique, distinct, ‘community’ support’ bringing familiarity to an unfamiliar, medically focussed environment. (2) Volunteers were able to ‘establish a connection centred on ‘being there’ within the acute hospital environment’ despite the fast paced and highly changeable environment. (3) Through ‘relational interactions adapted to the individual person’ volunteers attended to patients’ existential and emotional needs.

**Conclusion:**

These services confer benefits that are transferrable across cultures and countries, ‘fusing’ formal care with the informal visiting of family or friends, attending to patients’ existential needs. Recommendations include exploring ways to embed the end-of-life care volunteer service into this unique environment, alongside continuing research to explore cultural differences across different countries.

**What is already known about the topic?**

- Volunteering in palliative and end of life care has been shown to improve the holistic care of patients and families and improve wellbeing
- Volunteer services that provide direct support to patients receiving palliative and end of life care in hospitals are new and developing, but little is known about the use and experience of such services from key stakeholders.

**What this paper adds?**

- End of life care volunteers provide an opportunity to bring a ‘social’, or ‘community’, experience into the hospital environment
- The volunteers were in a unique position to complement the formal clinical care, due to their focus on the individual patient especially at the emotional and existential level
- The value of hospital end-of-life-care volunteer services lies in ‘compassionate community-based support’ for seriously ill patients within the context of a highly medicalised environment.

**Implications for practice, theory, or policy**

- Finding ways to recognise and formally integrate community-based volunteer services into the unique, highly medicalised environment of the acute hospital should be prioritised
- Further research to explore social and cultural differences is recommended.

## Background

Global estimates predict the number of people dying with serious health related suffering will increase from 26 million in 2019–48 million by 2060.^1^ This increase presents a challenge to existing healthcare systems to find innovative ways to optimise access to, and delivery of, palliative care, including psychosocial support,^1,2^ especially in the hospital setting where a significant number of people will still die.^3^ Often, dying in hospital is characterised as less than optimal,^4^ with patients dying ‘lonely deaths’ away from significant others and the familiarity of home.^5,6^ Hospitals are fast paced environments, and the demands of caring for multiple seriously ill patients may mean nurses are less able to provide necessary acts of care, such as providing comfort being present with their patients.^7,8^ However, there is also developing evidence to suggest that hospital can offer palliative care patients a place of ‘safety’.^9^ Considering that hospital will remain a common place of death for many people across the world, finding ways to enhance the care experience is essential, and the development of volunteer services may one such innovation.

Volunteers have a long tradition of providing vital support to palliative care services, most notably in the hospice sector.^10^ Volunteering is expected to play an even more substantial role in future palliative care in several countries,^11^ as such services have been shown to improve levels of satisfaction with care,^12,13^ offering a much-needed resource through providing social support, fulfilling surrogate family roles,^14,15^ and bridging the gap to the community.^16^ Volunteering in palliative care can also bring personal benefits for volunteers, such as a sense of commitment and connection to the people and place of volunteering, being valued as a caring person,^14,17^ and being able to make a difference in the lives of others.^18^ However, volunteer services that provide direct support to patients at the end of life in hospitals are less common, with limited research into their benefit, requiring further investigation to unpack specific issues related to volunteering within this unique setting, including exploration of experiences of delivering and receiving volunteer support.^19^

The iLIVE Volunteer Study (Clinical Trials registration: NCT04678310), one of eight work packages in the European Horizon 2020 Programme funded iLIVE Project,^20^ developed, implemented and evaluated end of life care volunteer services in five countries: the Netherlands, Norway, Slovenia, Spain, and the UK. Qualitative research was undertaken to explore the use and experience of these services in practice for key stakeholders. Data were collected between March 2021 and June 2023.

## Aim

Explore the experiences of volunteers and healthcare professionals, of a newly established hospital end-of-life-care volunteer service, in five countries.

## Method

A phenomenological approach was adopted within an interpretive research paradigm, focussing on subjective experiences and contextual factors to gain rich, detailed insights into social phenomena.

### The intervention: End-of-life care volunteer services

An end-of-life-care volunteer service was developed and implemented in each of the five participating hospitals by designated Volunteer Coordinators. The volunteer services were designed to provide support to seriously ill and dying patients in the hospital setting. Each site developed a bespoke volunteer training and service implementation plan, tailored according to the specific service needs of each country, based on the European Core Curriculum developed as part of this study.^21^ In three countries the end-of-life volunteer services were implemented within existing volunteer services as a discreet cohort of volunteers, but for two hospitals, the end-of-life-care volunteer service was the first volunteer service to be implemented within the care environment. Table 1 below provides an overview of the hospital end of life care services and the characteristics of each hospital in the five countries.

**Table 1.** Overview of the hospital end-of-life-care services and hospital characteristics in the five participating countries.

Hospital end-of-life care services and characteristics	The Netherlands	Norway	Slovenia	Spain	United Kingdom
Characteristics of hospital end-of-life-care volunteer services					
Number of active volunteers at launch of service	12	13	5	7	17
Total number of wards covered by the service	8	7	2	1	1
Characteristics of hospital					
Type of hospital	Large city centre teaching hospital	Large city centre teaching hospital	Tertiary clinic, Pulmonary and Allergic Diseases	Small general Hospital	Large city centre teaching hospital
Number of beds	600	1100	190	54	580 (old site)
					640 (new site – opened October 2022)
Does the hospital have an existing volunteer service active in the hospital?	Yes	Yes	No	No	Yes
Uptake of end-of-life care volunteer service					
Number of patients that accepted support from a volunteer	27	14	33	440	203
Total number of volunteer contacts for all patients	57	34	39	547	653
Average number of contacts per patient	2.1	2.4	1	1.2	3.2
Total time for all volunteer contacts (in minutes)	6255	4200	4392	20,160	30,110
Average time in minutes for each volunteer contact (mean)	110	123	113	37	46
Reason for volunteer contact					
Reading	3	0	0	2^a^	2
Talking	246	353	3	33^a^	22
Being present	21	265	8	39^a^	4
Eating	0	16	0	2^a^	-
Drinking	1	5	0	8^a^	-
Other	143	14	3	5^a^	4

a More than one reason was given for volunteer contact.

End-of-life care volunteer services were implemented within previously identified wards which the Volunteer coordinators determined as having higher proportions of patients with palliative and end of life care needs. The clinical team on the wards were responsible for identifying patients who may benefit from the service, and subsequently making a referral to the Volunteer Coordinator for allocation of a volunteer, if the patient agreed. Patients were identified as eligible for the volunteer service if they met the inclusion criteria for the iLIVE Volunteer Study; adult patients with capacity to give informed consent to participate, and where the clinical team answered ‘yes’ to an adapted ‘surprise question’, ‘would you be surprised if the patient were to die within the next month?’. Volunteer visits were one-to-one, face-to-face, at the patient’s bedside, and included activities such as: reading, talking, being present, eating, drinking. There was no limit to the number of visits that a patient could receive from a volunteer; repeat visits were determined on a patient-by-patient basis dependent on individual need, and within the capacity of the service. Table 1 provides a breakdown of the number of patients supported by the volunteer service, including number of visits per patient and activities engaged during visits.

#### Setting

This study was undertaken in the hospital setting, in five European countries. Table 1 provides an overview of the hospital end-of-life-care volunteer services, and the characteristics of the hospitals that they were implemented within, for the five participating countries.

#### Population

Participants were identified separately within each country, following protocols based on locally acquired ethical approvals. Participants were eligible for this study if they met the following inclusion criteria:

1. Adult Volunteers (<18 years) recruited and trained for the hospital end-of-life-care volunteer service developed as part of the iLIVE Volunteer Study.
2. Healthcare professionals, and allied healthcare professionals, working on the hospital ward(s) within which the end-of-life-care volunteer service was operating.

#### Sample

Volunteers from the end-of-life care volunteer services, and health care professionals from wards where the service was in operation, were recruited from within the individual participating countries by the local researchers. Potential volunteer and healthcare professional participants were first notified about the study by the Volunteer Coordinator for the end-of-life care volunteer service, who gained verbal consent to be contacted by a study researcher.

#### Recruitment

All participants were required to give their informed consent to take part in this study. The local study researchers provided potential participants with an information sheet to describe the study and invite them to take part in a focus group. If participants were happy to take part, then a consent form was signed prior to participation in the focus group.

#### Data collection

Focus group interviews were undertaken to explore the lived experience of the volunteer services from multiple perspectives, through interactive discussions.^22,23^ It is our assertion that phenomenology inherently involves ‘intersubjectivity’, in that meaning is jointly constructed, and individual experiences are situated within a wider social context and shaped by interactions with others.^24,25^ Focus groups encourage collaboration and dialogue between participants, which can illuminate the complexities of the ‘phenomenon’ under discussion through shared experiences and reflection.^22^

Focus groups were conducted separately with the two participant groups within each country. They were facilitated by members of the iLIVE Volunteer Study research team not directly involved in the operation or delivery of the volunteer service, except in one country (Slovenia) where the volunteer coordinator for the end-of-life-care volunteer service acted as the facilitator. Facilitators used a topic guide, to ensure relevant issues were covered, and encourage free discussion from the participants’ perspectives. During the focus groups, researchers made notes on the process and interactions between participants. Focus groups were audio recorded and transcribed verbatim for analysis.

## Analysis

An adapted framework analysis was used to structure the analysis within and across the different countries. Framework methodology is useful when members of the research team come from different disciplines and countries, and where the intention is to conduct an inductive, interpretive analysis.^26^ The analysis was led by an experienced qualitative researcher (AG), with experience of using framework analysis within international contexts, and where research data was collected in multiple languages requiring translation into English.^27^

### Development of the analysis framework and individual participant summaries

Authors AG and MB developed the analysis framework, based on the ‘topics’ covered within the topic guide (see supplementary information). Individual participant summaries were developed from the focus group transcripts, in English, by a nominated lead researcher in each country, under each topic in the analysis framework. Summaries included rich description of the participant perspective, from the interpretation of the local researcher. Summaries included descriptions relating to participant interactions where this was important for the analysis and where it enhanced the interpretation. Particularly for some of the volunteer focus groups, it was observed that some discussions among the participants generated insights and stories that may not have come out in a one-to-one interview, for example, through reminders of patient encounters or discussion regarding aspects of their roles.

### Analysis of participant summaries and development of themes

Participant summaries under each topic were analysed to develop themes. Researchers read through each participant summary, coding the data to identify similarities and differences between participant accounts, and identify concepts or ideas that were represented across the data set. Codes were interrogated to generate themes. Themes were refined through discussion between researchers allowing deeper interpretations. Researchers met online, with one face to face discussion mid-point through the analysis.

For non-English speaking countries, salient passages of text were translated into English, with support from the translation application DEEPL, so that selected quotes could be incorporated into the framework to illustrate concepts and ideas captured within that theme.

## Ethical approvals

Ethical approval was obtained in each participating country:

UK: Haydock Research Ethics Committee (reference: 20/NW/0271)
Netherlands: Medical Research Ethics Committees United (reference: R20.004)
Norway: Regional Committees for Medical and Health Research Ethics (Southeast) (reference: 35035)
Slovenia: The Commission of the Republic of Slovenia for Medical Ethics, (reference 0120-129/2020/3)
Spain: Ethics committee of the provincial investigation of Malaga (date issued: 24/09/2020)

## Findings

### Participants

There were 40 participants in total across the five countries, with 20 in the volunteer focus groups, and 20 in the HCP focus groups. Most participants were female (70%, *n* = 14/65%, *n* = 13). The HCP focus groups included mostly nurses (60%, *n* = 12), followed by healthcare assistants/auxiliary nurses (15%, *n* = 3), physicians (10%, *n* = 2), social workers (10%, *n* = 2), and a psychologist (5%, *n* = 1). Tables 2 and 3 provides a breakdown per country.

**Table 2.** Volunteer focus groups: Breakdown of participants.

Volunteer focus groups			
Country	Number of participants	Gender	*n*
The Netherlands	4	Male	1
		Female	3
Norway	3	Male	1
		Female	2
Slovenia	5	Male	0
		Female	5
Spain	4	Male	2
		Female	2
UK	4	Male	2
		Female	2

**Table 3.** HCP focus groups: Breakdown of participants.

HCP Focus Groups				
Country	Number of participants	Gender	*n*	Profession
The Netherlands	4	Male	1	Registered Nurse
		Female	3	Physician (*n* = 1), Registered Nurse (*n* = 2)
Norway	4	Male	2	Registered Nurse (*n* = 2)
		Female	2	Registered Nurse (*n* = 2)
Slovenia	3	Male	1	Registered Nurse
		Female	2	Social Worker (*n* = 1), Registered Nurse (*n* = 1)
Spain	5	Male	2	Psychologist (*n* = 1)
		Female	3	Social worker (*n* = 1), Auxiliary Nurse (*n* = 1), Registered Nurse (*n* = 1)
UK	4	Male	1	Physician
		Female	3	Healthcare Assistant (*n* = 2), Registered Nurse (*n* = 1)

### Themes generated from analysis of focus groups

Three themes were generated: (1) Unique, distinct and community-based support in the hospital setting, (2) Establishing a connection and the value of ‘being there’ within the acute hospital environment, (3) Relational interactions adapted to the individual person. These are presented below. The perspectives of volunteers and healthcare professionals are presented separately, so that the distinct viewpoints of each participant group are highlighted within the narrative.

*1. Unique, distinct, ‘community-based support’ in the hospital setting*

#### Volunteers

Volunteer accounts revealed a role that was distinct and separate from formal clinical care on the ward. Their encounters with patients included sitting, listening, talking about hobbies, interests, and sharing stories about their lives outside the confines of the hospital. Volunteers often framed these activities as ‘just usual/nothing out of the ordinary’, for example, ‘I just listened’ (Vol ENG-2); however, being able to provide this for patients was their unique benefit, bringing the ‘normality’ and ‘familiarity’ of community into an unfamiliar environment. This can be seen in the excerpt below in which a volunteer describes one encounter with a patient which had the feel of a visiting friend.

*“I brought a bottle of pop in for [a patient] the other day, a bottle of Cream Soda, he drank that and then I thought eh…so I bought another one [for me and him]…create some kind of relationship” [Vol UK-1]*

The training programme helped volunteers to prepare for engaging in this ‘community-based support’ and provided them with ‘realistic’ expectations about what being an end-of-life-care volunteer would entail, including practical examples around how to engage in these ‘usual’ activities with patients and families. Some volunteers reported that clinical staff were apprehensive, or uncertain, when they were initially initiated onto the wards, but awareness of the comprehensive training programme ameliorated clinician concern.

*“What I think is the biggest barrier, what I notice, is that [staff on the wards]…ask what our professionalism is, what we can do, how we were trained, what we do. And when I tell them that we took a whole course, that we really went to great lengths to ensure that we were properly supervised and trained, they are really wide-eyed.” [Vol NL-2]*

#### Healthcare professionals

Volunteers provided support as an adjunct to the clinical care provided by the ward staff, which healthcare professionals saw as enhancing the overall quality of care patients received. Their accounts highlighted the benefits of the ‘community-based support’ provided by volunteers, especially for patients with limited family visitors or social contact whilst in the hospital. One healthcare professional reported a direct personal experience of the end-of-life-care volunteer service when her mother died. Her reflection, in the excerpt below, was from a personal perspective of being supported by the volunteer whilst being a visiting relative when her mum was dying, and describes how the volunteer acted as a ‘bridge’ between the clinical staff and even her family members.

*“I think they are valuable from a personal point; you know what I mean, [rather] than talk to your own family you can sort of open up to them…she [volunteer] used to have us in here and have a little chat and comfort us, you know, she knew all the right words…” [HCP UK-04].*

Some healthcare professionals reported uncertainties about the role and purpose of the volunteer, including role boundaries. However, knowing the comprehensive training the volunteers received helped them to see that the role was distinct, rather than an attempt to supplant the care provided by the clinical team.

*“What I was also a bit apprehensive about beforehand was who will be sitting at that bedside? And what I found very reassuring is that the volunteers all had that training, and I did have very good experiences with that.” [HCP NL-4]*

*2. Establishing a connection centred on ‘being there’ within the acute hospital environment*

#### Volunteers

Volunteer accounts revealed a defining characteristic of the role centred on ‘being there’ by establishing connections to build a ‘social bond’, even if a patient was unable to communicate verbally, was semi-conscious or unconscious, or time was short. Some used clues from the immediate environment to locate points of shared history or experience, such as artefacts that signified a religious interest or belief, or other items or pictures in the patient’s room or bedside on which to base their interaction and develop a personal link.

*“…most of [the patients] would be unconscious so you would be only going in there, and you would just have to try and make up a conversation you know. I also remember there was a little man sitting there, lying there unconscious and I spoke to him and things like that and his son came and his son said, started talking to me and I found out this little old man had been, about 19 years of age, had been flying Lancaster Bombers over Germany. You know you can’t, you can’t really think of that can you.” [Vol UK-2]*

Some volunteers were reliant on ward staff to facilitate the initial introduction between themselves and the patient. When this was not as smooth as it could be, the connection with the patient could be compromised, highlighting the importance of a good first impression.

*“One thing, for example, for the medical staff. Maybe it’s not so important and maybe I have to tell the health worker that it’s very important to me. And it happened to me that they can’t make a mistake in giving me the name of the person. I can’t go in, Pepita, how are you? And look at me with a face like a cut-up gazpachuelo [surprise] and tell me I’m not Pepita. Of course, your face is like an impersonal face. I mean, I’m going in and I’m not calling the lady by her name. And then we realised that the staff had made a mistake” [Vol SPA-1]*

In some of the hospitals the volunteers were re-introduced after COVID-19 when social distancing measures were eased, but relatives were still unable to visit patients. In this situation volunteers were able to ‘be there’ and provide human contact and companionship during a potentially lonely and emotional time for the patients.

*“During the time of COVID, we were somehow more needed. People truly needed that contact, even in instances like when I assisted patients outside because we couldn’t meet otherwise.” [Vol SLO-4]*

The ‘activities’ volunteers engaged in with patients reflected a relational and emotional orientation, helping to facilitate a human ‘connection’ with patients (and families). In this situation, connection rather than action was the focus. Building connections by offering to ‘be there’, or ‘simply’ listening, had catalysing benefits, with volunteers describing occasions where patients opened up about concerns, or issues worrying them, that they hadn’t been able to share with friends or family.

*“[patients would] be spilling their problems out to you. We weren’t allowed to give advice, we were only able to lend a listening ear you know, but it’s amazing, it was amazing how some of these people, after they had finished talking to me and all that, they would say [to me] thanks very much for your help and I would be going, I just listened.” [Vol UK-1]*

#### Healthcare professionals

Healthcare professionals described how volunteers were able to connect to patients in a different way than they could as ward staff, mainly due to the dedicated time, enabling ‘one on one’ attention. Due to busy wards, or low staffing levels, healthcare professionals were unable to guarantee individualised attention and valued the relationships and connections they observed between volunteers and their patients. However, the context of acute hospital environment could limit opportunities for volunteers to establish connections. For example, patients are often very sick, requiring lots of interventions, compounded by the often-rapid turnover of patients in the hospital environment and one-off nature of support.

*“The rapidly changing conditions of patients are a barrier, and their condition often changes from day to day. Sometimes it is difficult to coordinate volunteers and patients because patients have been [sent] home before the visit takes place.” [HCP SLO-1]*

Despite these challenges, many healthcare professionals observed meaningful connections conferring benefits to patients such as providing company, offering a brief distraction from their worries, or even reducing anxiety. Even when encounters were short, they saw volunteers find ways to meaningfully connect with patients.

*“I have had very good feedback that those [volunteers] who have engaged have engaged a lot. In fact, I have felt that it has even helped my interventions, because I find the patient very relaxed.” [HCP SPA-1]*

One healthcare professional from the UK described a specific example where the connection and presence provided by the volunteer aided in building trust between the patient and clinical staff.

*“[I remember] when the volunteers joined the team, we had a very difficult gentleman who had no trust in any of us and…I think the volunteers sitting with gentleman, and was (sic) talking about his interests which we didn’t have the time to sit down and talk about, gardening, which is what he loved doing, the volunteers were able to do that.” [HCP UK-02]*

Having someone to ‘be there’ for patients also had practical benefits for healthcare professionals. For example, being in their room or at the bedside provided an extra, much-needed, pair of ‘eyes and ears’, alerting staff to changes in the patient’s condition requiring urgent attention. This was a highly prized commodity for many of the clinical staff, particularly when framed in the context of a busy ward environment and competing priorities prohibiting them from ‘being there’ all the time. The presence of the volunteer provided comfort to staff as well as patients, and gave them confidence that should they be needed, the volunteer would be on hand to let them know.

*“I experienced it as very pleasant because it gave me the feeling, I did not have to go inside the room all the time out of fear that the patient would otherwise be lonely. So, for me it was a pleasant addition.” [HCP SLO-3]*

*3. Relational interactions adapted to the individual person*

#### Volunteers

Volunteer involvement potentiated patient interaction and support that was ‘person-centred’ and responsive. The ability to establish a connection with patients helped them to identify what was important to this unique person, to focus solely on their needs, wishes, or concerns by being physically present at the bedside without distraction. This enabled them to be active listeners, therefore able to offer moral support, comfort, and personalised attention; the ‘little things’ that volunteers did to make the connection enabled them to attune to what mattered for that person.

*“You’re there literally sitting there…you know just relax that sort of thing, you know just to, just gently just to talk to them every few minutes or just to making sure you are there sort of thing.” [Vol UK-3]*

The social aspect of the role was highlighted as a central benefit. Volunteers’ accounts revealed the importance of having the skills to identify patients who needed company, to relieve boredom or loneliness in the hospital environment if family were unable to visit, or support families that needed respite.

*“I spoke several times with the relatives, and I sincerely feel sorry for them. But they said, clearly expressed that they were, they were tired. And you could see that, too. They were very grateful that we were there.” [Vol NOR-1]*

Providing this type of intensely individual support can take an emotional toll. Being open and emotionally available for patients during a potentially sensitive, complex or distressing time was challenging, requiring volunteers to be attuned to their own needs, to cope with intense interactions around the bed. Finding ways to cope with the emotional load was important and was facilitated by the ongoing supervision sessions led by the volunteer coordinators or managers of the services.

*“Sitting, being there, that has been very tiring for me in all cases. Because I was always there for a very long time. Three hours sometimes in a row…with people who were comatose, had no reaction at all, and then sitting for three hours is very long. I also sometimes came in with someone who said I’m glad you’re here and he started his conversation, which also lasted three hours. Which I didn’t have to participate in at all and that was just as exhausting.” [Vol NL-1]*

#### Healthcare professionals

For healthcare professionals, seeing the individualised care volunteers were able to provide positively influenced their understanding and appreciation of what the volunteers could offer patients. The volunteers are ‘complementary’ to the clinical care delivered by the ward staff, placing them in a unique position to provide much needed individualised support, ‘free from the demands of the ward’ (HCP ENG-2), able to provide one on one, dedicated attention, without competing priorities.

*“Some [patients] are very lonely, very confused and scared people and having no one there in an environment like this, even on this ward, it can be an absolutely terrifying experience…So just having…someone who can sit there and give some people constancy[sic] through the day, just a friendly face, even just a reassurance that if there’s a problem they can tell them and they can go and fetch a nurse, rather than have to wait for ages for the buzzer for example. And I think sometimes it’s been a great help because we are able to reassure the families of our patients that, look, we know you don’t want mum to be alone, for example, but why don’t you go and get a cup of tea we’ve got a volunteer they will sit with mum, she won’t be alone etc, so it takes a lot of pressure off families as well.” [HCP UK-1]*

Healthcare professional accounts also revealed the emotional resilience volunteers required, to provide individualised, close contact care for dying patients in the hospital. For example, one nurse explicitly stated that they *‘stress out’ [HCP SLO-3]* to think of what it takes to prepare a volunteer for this kind of role. Healthcare professionals perceived the role as specialist, requiring specific training and support, and perhaps not suited to all volunteers; one nurse from Slovenia questioned whether there were some people inherently more suited to the role.

*“It’s probably a psychological trait among volunteers; I imagine that they don’t approach all patients in the same manner, that they tread a bit cautiously, and know when [to] joke and when [you wouldn’t].” [HCP SLO-2]*

## Discussion

### Main findings

Hospital end-of-life-care volunteers are distinct, separate, and complementary to formal caring roles within the hospital. A unique benefit of the end-of-life-care volunteer was their ability to ‘be there’ for patients in ways that the clinical staff could not, due to competing demands of the busy hospital environment. They were able to provide meaningful company and comfort to patients if family were unavailable, although many volunteers also supported patients alongside family members or friends.

The activities volunteers engaged in reflected a relational and emotional orientation, bringing familiarity into an unfamiliar medically focussed environment and facilitating human connections. The busy and frenetic hospital environment, alongside the often-deteriorating clinical situation of the patient, emphasised the importance of being able to make meaningful connections especially where time may be short. The complementary nature of volunteering created the potential for interaction and support that was responsive to the ‘whole person’, especially at the emotional and existential level. For clinical staff, having a clear understanding of the role of the volunteer, as well as how they have been trained to undertake that role, could promote more cohesive working relationships with the volunteers, allay concerns, and enhance the service provided to patients.

### What this study adds

Previous research suggests volunteers in palliative care occupy a liminal space between professional and family roles that is distinct and different to formal patient care, focussed on psychosocial and existential needs,^11^ ‘bridging a gap’ between the secluded hospice environment and the community through their ‘normalising role’.^16^ These findings support and explicate this for the hospital setting, by suggest that volunteers present an opportunity to bring a ‘social’, or ‘community’, experience back into the highly routinised and medicalised clinical environment,^28^ situating them as a ‘community resource’ located in the hospital, rather than a component of the healthcare team. These findings highlight that volunteers were in a unique position to complement, and not just ‘support’, the formal clinical care on the ward, which was reflected across all services within this study. For volunteers, being able to find points of shared interest or history due to their links with the local community enhanced connections with patients. They were free to adapt their support through varied and individualised interactions, shaped by the cultural and social context, without being assimilated into the ‘professional’ culture of the ward.^29,30^ The significance of this is increased in the hospital setting, where patients are separated from their communities, and acutely unwell with an often-rapidly deteriorating physical condition. As such, these findings also highlight that the propensity to situate volunteer activities within the ‘language and rules’ of formal clinical care^27,28^ neglects to meaningfully encapsulate their contribution, and risks marginalising their role in the hospital as merely ‘supporting’. These findings suggest a necessity to define and demarcate this significant role, and argue that the value of hospital end-of-life-care volunteer services lies in the harnessing of, what Kellehear calls, a ‘compassionate community’ model,^31^ for seriously ill and dying people and their families, within the context of a highly medicalised environment. Additionally, attention to cultural nuances within the volunteer service, and the patients and families they support, ensures that the service is tailored to local contexts.

Varying role responsibilities, coupled with wide variations in training provided to palliative care volunteers, especially in the hospital setting,^19^ can contribute to staff unease regarding involving volunteers.^14^ Perhaps due to the novelty of the service, a minority healthcare professionals in this study reported boundary concerns regarding what specific activities the volunteer should be engaging in, as well as concerns regarding the emotional impact of supporting dying patients.^32^ However, this study also found that where staff had more awareness of the training content and the role of the volunteer, they were more accepting of the volunteers in the environment, which directly supports the supposition from Scott et al. that educating healthcare staff about the unique contribution of volunteers can enable such services to flourish.^27^ It has also been argued that hospice volunteers benefit from a sense of ‘belonging’, and that good training and support within the organisation are essential to optimise this, ensuring an environment where volunteers can contribute meaningfully.^17^ Volunteers in this study received comprehensive training that included attention to the unique environment of the acute hospital setting, cultivating a relationship between volunteer and clinical staff, developing coping strategies, and reflective practice.^21^

Palliative care patients, and patients towards the end of life, are more at risk of social isolation and feelings of loneliness,^33–35^ which can be exacerbated by being in a busy, unfamiliar medical environment such as the hospital.^8^ It has been argued that interventions, or services, which offer social, or emotional, support to palliative care patients could impact significantly on feelings of depression and improve perceptions about quality of life.^36^ Findings from this study suggest that the relational and existential support offered by the hospital end-of-life-care volunteer services may be one way to address this. This was a unique and different kind of support in the hospital environment, fusing aspects of formal care with the informal visiting of family or friends,^28^ and with attention to the patients’ existential needs, argued to be a neglected aspect within healthcare policies.^37^ In this study, volunteers had the ‘gift of time’ for patients and could be attentive to patients’ individual needs without competing clinical priorities.^7,8^

### Strengths and limitations

Five locally adapted hospital end-of-life-care volunteer services were successfully developed in five countries, underpinned by a European Core Curriculum developed as part of the iLIVE Volunteer Study.^21^ Membership of the iLIVE Consortium promoted collaboration between researchers at each stage of the project, across different settings and cultures. Focus groups elicited rich descriptions of lived experiences, and the adapted framework approach promoted collaborative analysis of data across five different languages, optimised using individual participant summaries which enhanced discussion during the analysis phase. Nevertheless, our study has some limitations. It included a small number of participants with experience of the five services, meaning the findings may not be transferrable to other services within different contexts. Similarly, although this study included participation from five European countries, findings cannot be interpreted as a ‘European perspective’, nor can they discern specific cultural or country-specific differences between services.

## Conclusions

Finding ways to recognise and formally integrate and embed these volunteer services into the unique, highly medicalised environment of the acute hospital should be prioritised, to ensure that dying patients receive holistic support that addresses their existential and emotional needs alongside clinical care. Further research should continue to explore cultural differences within the end-of-life-care volunteer role across different countries. Findings from this study may inform and inspire other hospitals, and countries, to consider how end-of-life-care volunteers may improve care delivery.

## Supplemental Material

sj-docx-1-pmj-10.1177_02692163251328197 – Supplemental material for iLIVE volunteer study: Volunteer and healthcare professional perceptions of newly developed hospital end-of-life-care volunteer services, in five countries Supplemental material, sj-docx-1-pmj-10.1177_02692163251328197 for iLIVE volunteer study: Volunteer and healthcare professional perceptions of newly developed hospital end-of-life-care volunteer services, in five countries by Tamsin McGlinchey, Stephen Mason, Grethe Skorpen Iversen, Dagny Faksvåg Haugen, Inmaculada Ruiz Torreras, Pilar Barnestein Fonseca, Miša Bakan, Berivan Yildiz, Ruthmarijke Smeding, Anne Goossensen, Agnes van der Heide and John Ellershaw in Palliative Medicine

sj-docx-2-pmj-10.1177_02692163251328197 – Supplemental material for iLIVE volunteer study: Volunteer and healthcare professional perceptions of newly developed hospital end-of-life-care volunteer services, in five countries Supplemental material, sj-docx-2-pmj-10.1177_02692163251328197 for iLIVE volunteer study: Volunteer and healthcare professional perceptions of newly developed hospital end-of-life-care volunteer services, in five countries by Tamsin McGlinchey, Stephen Mason, Grethe Skorpen Iversen, Dagny Faksvåg Haugen, Inmaculada Ruiz Torreras, Pilar Barnestein Fonseca, Miša Bakan, Berivan Yildiz, Ruthmarijke Smeding, Anne Goossensen, Agnes van der Heide and John Ellershaw in Palliative Medicine
