# Supplementary material for: iLIVE volunteer study: Volunteer and healthcare professional perceptions of newly developed hospital end-of-life-care volunteer services, in five countries
Source: Palliat Med. 2025 May 29;39(7):792–802. doi: 10.1177/02692163251328197 (PMC12227806; doi:10.1177/02692163251328197)
Supplement: sj-docx-1-pmj-10.1177_02692163251328197 – Supplemental material for iLIVE volunteer study: Volunteer and healthcare professional perceptions of newly developed hospital end-of-life-care volunteer services, in five countries [file sj-docx-1-pmj-10.1177_02692163251328197.docx]

Palliative and End of Life Care Volunteer – Focus Group Topic Guide

**Volunteers who have provided support for at least 1 patient who has been referred to the hospital palliative and end of life care volunteer service (mid way through the data collection period).**

Focus groups will be approximately 1 hour, however this will be determined by the group. There will be a ‘light’ structure to the focus group, to allow open discussion and ensure that topic areas are covered. Participants will be encouraged to talk freely about their experience of the service and any benefits that they perceive for patients/families, or any negative effects.

The focus group will be based on the following ‘schedule’:

Time: 1 hour

| 10 | Introductions  Consent |
| --- | --- |
| 20 | Could you tell me what motivated you to become an iLIVE volunteer?  Have you done any previous volunteering and how did this experience differ?  What were your expectations of what being an iLIVE volunteer would be and were these expectations met? |
| 20 | Specific experiences:   - What was your experience of ‘being with’ patients who may die whilst in hospital? - How did you feel about the prospect of having a patient die whilst you were providing support for them? - Did you ever expect any patients to die whilst you were with them? - How would you describe the impact you have on patients or their families? - What went well? - What could have gone better? - Would there be any situation in which you would not feel it was appropriate to be with a patient at this time? - What if any, are the main barriers to the iLIVE volunteers within the hospital setting? |
| 10 | Final close:  Based on your experiences, how would you improve the volunteer service?  (e.g. access to volunteers, role of volunteers, length of involvement with patients etc) |
