# Supplementary material for: iLIVE volunteer study: Volunteer and healthcare professional perceptions of newly developed hospital end-of-life-care volunteer services, in five countries
Source: Palliat Med. 2025 May 29;39(7):792–802. doi: 10.1177/02692163251328197 (PMC12227806; doi:10.1177/02692163251328197)
Supplement: sj-docx-2-pmj-10.1177_02692163251328197 – Supplemental material for iLIVE volunteer study: Volunteer and healthcare professional perceptions of newly developed hospital end-of-life-care volunteer services, in five countries [file sj-docx-2-pmj-10.1177_02692163251328197.docx]

Healthcare professional – Focus Group Topic Guide

**Healthcare professionals who have cared for at least 1 patient who has received support from at least 1 iLIVE volunteer ‘episode’ (mid way through the data collection period)**

Focus groups will be approximately 1 hour, however this will be determined by the group. There will be a ‘light’ structure to the focus group, to allow open discussion and ensure that topic areas are covered. Participants will be encouraged to talk freely about their experience of the service and any benefits that they perceive for patients/families, or any negative effects.

The focus group will be based on the following ‘schedule’:

Time: 1 hour

| 10 | Introductions  Consent |
| --- | --- |
| 20 | Could you tell me, from your perspective as a *staff member*, what did you understand about the involvement of the volunteer?  What were your expectations of what the volunteer would be able to do for patients who are in the last month of life and were these expectations met? |
| 20 | Specific experiences:   - How did having a volunteer to ‘be with’ your patient(s) make you feel? - How do you feel that the volunteer impacted on the care patients receive, or their experience overall? - What went well? - What could have gone better? - Would there be any situation in which it would not be appropriate to have a volunteer be with a patient? - What if any, are the main barriers to the volunteers within the hospital setting? |
| 10 | Final close:  Based on your experiences, how would you improve the volunteer service?  (e.g. access to volunteers, role of volunteers, length of involvement with patients etc) |
